# The Effects of Xiangqing Anodyne Spray on Treating Acute Soft-Tissue Injury Mainly Depend on Suppressing Activations of AKT and p38 Pathways

**DOI:** 10.1155/2016/9213489

**Published:** 2016-04-14

**Authors:** Shudong Wang, Tao Li, Wei Qu, Xin Li, Shaoxin Ma, Zheng Wang, Wenya Liu, Shanshan Hou, Jihua Fu

**Affiliations:** ^1^Department of Pharmaceutics, Jinling Hospital, Nanjing University School of Medicine, Nanjing 210002, China; ^2^China Pharmaceutical University, Nanjing 211198, China; ^3^Department of Physiology, China Pharmaceutical University, 639 Long Mian Road, Nanjing, Jiangsu 211198, China

## Abstract

*Objectives*. In the present study we try to elucidate the mechanism of Xiangqing anodyne spray (XQAS) effects on acute soft-tissue injury (STI).* Methods*. Acute STI model was established by hammer blow in the rat hind leg muscle. Within 8 hours, instantly after modeling and per 2-hour interval repeated topical applications with or without XQAS,* CP* or* IH* ethanol extracts spray (CPS and IHS) were performed, respectively; muscle swelling rate and inflammation-related biochemical parameters, muscle histological observation, and mRNA and protein expression were then examined.* Results*. XQAS dose-dependently suppressed STI-caused muscle swelling, proinflammatory mediator productions, and oxidative stress as well as severe pathological changes in the injured muscle tissue. Moreover, CPS mainly by blocking p38 activation while IHS majorly by blocking AKT activation led to cytoplastic I*κ*B*α* degradation with NF-*κ*B p65 translocated into the nucleus. There are synergistic effects between* CP* and* IH* components in the XQAS on preventing from acute STI with suppressing I*κ*B*α* degradation, NF-*κ*B p65 translocation, and subsequent inflammation and oxidative stress-related abnormality.* Conclusion*. Marked effects of XQAS on treating acute STI are ascribed to strong anti-inflammatory and antioxidative actions with a reasonable combination of* CP* active components, blocking p38-NF-*κ*B pathway activated, and* IH* active components, blocking AKT-NF-*κ*B pathway activated.

## 1. Introduction

There are many ancient archives for treatment of soft-tissue injury (STI) using Traditional Chinese Medicine. STI care can be traced back to early civilizations, and many of these treatments were based on the use of herbal remedies. The report showed that approximately one-third of all traditional medicines in use are for the treatment of wounds and soft-tissue disorders, compared to only 1%–3% of modern drugs [1]. Xiangqing anodyne spray (XQAS) is a spray formulation by topical administration, which is a mixture of two ethanol extracts that were extracted, respectively, from equal-mass crude of two Traditional Chinese Medicines,* Cynanchum paniculatum* (*CP*) and* Illicium henryi* (*IH*), with additional penetration enhancers. In previous study, we have found that XQAS effects on treating neuralgia and postherpetic neuralgia in clinical application were prominent and superior to the ethanol extract of* IH* or* CP* treatment alone [2, 3]. In the modern medicine, the ethanol or water-soluble extracts of* CP* have been demonstrated to relieve various pains, such as rheumatic arthralgia, lumbago, pain due to traumatic injuries, abdominal pain, and toothache, which were also used to treat skin diseases such as eczema, rubella, and neurodermatitis [4]; the water-soluble extracts of* IH* have also been applied clinically as an analgesic agent by intramuscular injection in China [5]. We found that paeonol and quercetin may be the major active compounds in* CP* and* IH* (see Section 2), respectively. Paeonol effects on anti-inflammatory, antioxidant, and cardiovascular protective activities have been demonstrated [6], while antioxidant properties of quercetin associated with anti-inflammatory effects and so forth were also reported [7, 8].

The acute STI, majorly showing skeletal muscle injury, is mainly traumatic aseptic inflammation. When it occurs, the pathological changes include local tissue necrosis, blood capillary dilation, inflammatory cell infiltration with release of inflammatory mediators, and tissue edema [9]. Further, the secondary lesion growth is related to progressive microcirculatory and inflammatory reaction. Direct trauma to microvessels results in membrane damage, endothelial cell swelling, widely vascular hemorrhage, and uncontrolled clot formation, leading to additional local ischemia, which, in turn, induces further degradation of membrane phospholipids, accumulation of free radicals, and inflammation-induced oxidative stress [10, 11]. As the extension of injury process, severe muscle fibers degeneration and necrosis following marked collagen fiber hyperplasia, massive inflammatory cell infiltrates, and interstitial ecchymosed will emerge in the site of damaged tissue to create irreversible structural changes [12]. Hence, early diagnosis and treatment with rapid attenuating tissue swelling and inhibiting inflammation can improve functional results and diminish structural damage in the early and acute period of skeletal muscle injury, which is necessary for treatment of STI [13]. According to the mechanism of inflammatory production, nuclear factor-*κ*B (NF-*κ*B) directly participates in inflammation reaction [14], while both MAPK and AKT signaling activation, in turn, activates transcription factors, such as activator protein-1 and NF-*κ*B, which promotes the gene and protein expressions of inflammation-correlated cytokines [14, 15].

Our previous study has indicated that repeated topical administration with XQAS in the STI site, which was modeled by hammer blow in the hind leg of rat, showed rapidly therapeutical effects on soft-tissue swelling and inflammatory mediators production, while repeated XQAS treatment for four days significantly suppressed STI-caused skeletal muscle necrosis and fibrosis, effectively recovering impaired muscle tissue in a dose-dependent manner. There is a synergistic effect between* CP* and* IH* in the XQAS [16]. XQAS effects were associated with suppression of activated NF-*κ*B p65 gene expression in the impaired muscle tissue. However, the molecular mechanism of XQAS effects, especially the synergistic effect between* CP* and* IH* on rapidly treating STI, remains unclear. This study tries to elucidate the mechanism of prominent efficacy of XQAS on treating acute STI and the reason why the efficacy is superior to* CP* or* IH* alone. An acute STI model with mass-drop injury method, according to Stratton et al. introduction [17], was constructed in this study.

## 2. Material and Methods

### 2.1. Preparation of Xiangqing Anodyne Spray (XQAS)

XQAS was prepared as previously described [16]. Briefly, the root barks of* Cynanchum paniculatum* (*CP*) or* Illicium henryi* (*IH*) were extracted by 75% ethanol, and penetration enhancers, which include Azone, peppermint oil, and PEG 400 borneol in the 75% ethanol, were added. The final XQAS contains 0.50 g (crude drug)* CP* and 0.50 g (crude drug)* IH* per milliliter, which contains 0.858 mg paeonol and 7.33 mg quercetin per milliliter. XQAS was examined by high performance liquid chromatography analysis. Using the same method, the* Cynanchum paniculatum* spray (CPS) and* Illicium henryi* spray (IHS) were prepared, containing 1.0 g (crude drug)/mL* CP* or* IH*, respectively.

### 2.2. Animals

The animal experiment was performed in accordance with China state regulations on animal experimentation and approved by Animal Experimental Ethical Center of Southeast University (protocol number 20120023). Male Sprague-Dawley rats (250 to 300 g) were supplied by Suzhou Industrial Park, Matt Ireland Ltd. All animals were maintained on a standard 12 h light-dark cycle, in a temperature-controlled environment (24 ± 2°C), with free access to water and chow. They were acclimatized for at least one week before initiating the experiments.

### 2.3. Acute Closed Soft-Tissue Injury (STI) Induction

Like the previously described method [16], the rats were anesthetized by ethyl ether, the inner hind thigh was epilated with depilatory Na_2_S solvent in advance and fixed in lateral, and the thigh muscle was hit with a cylindrical hammer (200 g in weight, 1.0 cm in top diameter, and 1.5 cm in bottom diameter) by free falling vertically from inside of a hard smooth plastic tube (75 cm in length and 1.5 cm in inner diameter), only resulting in closed STI but not to cause femoral fracture, which was established by a drop-mass method, similar to the model first described by Stratton et al. [17].

### 2.4. Animal Experiments

The rats were randomly divided into six groups (*n* = 8 per group): control (Ctrl), model (Mod), high-dose XQAS treatment (HXQAS), low-dose XQAS treatment (LXQAS), CPS treatment (CPS), and IHS treatment (IHS). Except for Ctrl group, other-group rats were subject to two-time blows to hind leg muscle to model acute STI. After modeling, the rats were instantly applied topically with 150 *μ*L/time penetration enhancers in the Ctrl and Mod group, XQAS in the HXQAS (containing 0.50 g (crude drug)/mL both* CP* and* IH*, resp.) and LXQAS (containing 0.25 g (crude drug)/mL both* CP* and* IH*, resp.) group, CPS (containing 1.0 g (crude drug)/mL* CP*), or IHS (containing 1.0 g (crude drug)/mL* IH*), respectively. Next, topical application was repeated with a 2-hour interval in a duration of 8 h after modeling, and the circumference located in the injured thigh muscle was measured at 0 (before modeling), 1, 2, 3, 4, 6, and 8 h; muscle swelling rate (MSR) was then calculated (MSR (%) = (*S*/*S*_0_ − 1) × 100%, where *S*_0_ represents the circumference at 0 h, while *S* represents the circumference at the different time point after making the model) [18]. At the end of experiment, rats were sacrificed after anesthetization with urethane (1.0 g/kg), and the injured thigh muscle in each rat was then divided into two parts: one was stored at −80°C to be used to measure biochemical parameters and to detect gene or protein expressions; the other was fixed in neutral buffered formalin to be used to observe the morphological change.

### 2.5. Biochemical Analysis of the Muscle Tissue

The muscle tissue was homogenized in 0.02 M phosphate buffered solution (PBS, pH 7.4). The parameter was measured according to the protocols of respective kits. The levels of interleukin-1 beta (IL-1*β*), tumor necrosis factor-alpha (TNF-*α*), and prostaglandin-E2 (PGE-2) were measured by ELISA kits provided by Abcam (HK) Ltd. The contents of myeloperoxidase (MPO), nitric oxide (NO), malondialdehyde (MDA), and superoxide dismutase (SOD) were measured by spectrophotometer kits, respectively, provided by Nanjing Jiancheng Bioengineering Institute.

### 2.6. Reverse Transcription-Polymerase Chain Reaction (RT-PCR) Analysis

The muscle tissue in six of the eight rats in each group was randomly chosen for RT-PCR assay. Total RNA was isolated from tissues using RNAiso plus Isolation Reagent (TAKARA, Otsu, Shiga, Japan). Total RNA solution was first reverse transcribed and then immediately amplified in a GeneAmp PCR system (Eppendorf). Primers used were NF-*κ*B p65 (forward-GGG ACT ATG ACT TGA ATG CG; reverse-CAG GCT AGG GTC AGC GTA T), IL-1*β* (forward-GAT GAC GAC CTG CTA GTG T; reverse-CTT CTT CTT TGG GTA TTG TT), TNF-*α* (forward-TCC AGG CGG TTG CCT ATG T; reverse-GAG CGT GGT GGC CCC), and GAPDH (forward-ATG TAT CCG TTG TGG ATC TG; reverse-GAT GGT ATT CGA GAG AAG GG). The gel was photographed by GeneGenius automatic gel imaging and analysis system (Syngene, Cambridge, UK) and the bands on the film were scanned by densitometry for quantitation. To exclude variations due to RNA quantity and quality, the data for all genes were adjusted to GAPDH.

### 2.7. Western-Blot Analysis

Six randomly chosen injury muscle samples from each group were homogenized in lysis buffer (150 mM NaCl, 10 mM HEPES, pH 7.9, 1 mM EDTA, 0.6% NP-40, 0.5 mM PMSF, 1 *μ*g/mL leupeptin, 1 *μ*g/mL aprotonin, and 10 *μ*g/mL trypsin inhibitor). Samples were then sonicated and incubated on ice for 15 minutes. Protein was extracted by Cytosol and Nucleus Protein Extraction Kit (KeyGEN Bio TECH). Protein concentration was determined by Bradford's method. Samples were separated by SDS-PAGE gel and electrophoretically transferred to nitrocellulose membrane. Nonspecific binding sites were blocked with Trisbuffered saline (TBS; 40 mM Tris, pH 7.6, 300 mM NaCl) containing 5% BSA for 12 h at 4°C. And the membranes were incubated with anti-NF-*κ*B (p65), anti-COX-2, anti-iNOS, anti-I*κ*B*α*, anti-phosphor-AKT (Ser^473^), anti-AKT, anti-phosphor-p38 (Thr^180^/Tyr^182^), anti-p38, anti-phosphor-JNK (Thr^183^/Tyr^185^), anti-JNK, anti-*β*-actin, and anti-Lamin B1 antibodies (all from Signalway Antibody Co., Ltd., USA) at 1 : 1000 concentration overnight at 4°C. After being washed with TBST (TBS with 0.1% Tween-20), membranes were incubated with secondary antibody for 2 h at room temperature, which was conjugated to horseradish peroxidase (Santa Cruz Biotechnology, Inc., Santa Cruz, CA). Immunopositive bands were visualized by a chemiluminescent method (ECL, Tanon-5200).

### 2.8. Histological Observation of Muscle Tissue

After fixation in neutral buffered formalin, the muscle was sectioned and processed routinely for hematoxylin-eosin (HE) staining for qualitative histological analysis. All histopathological examinations were performed by a trained pathologist. The muscle injury, including mainly muscle fibers degeneration and necrosis, interstitial ecchymosis, and inflammatory cell infiltrates, in each sample was assessed using the method reported by Bunn et al. [13].

### 2.9. Statistical Analysis

Results were expressed as mean ± standard deviation (SD). For statistical significance, except the MRS differences between groups which were tested using analysis of covariance (the baseline value in the control as a covariate), the other data were tested using one-way analysis of variance (ANOVA) followed by LSD's multiple comparison test. Differences were considered significant at *P* < 0.05 and extremely significant at *P* < 0.01.

## 3. Results

### 3.1. The Effects of XQAS on Muscle Swelling Rate (MSR) in Acute STI Model

**Figure 1:**
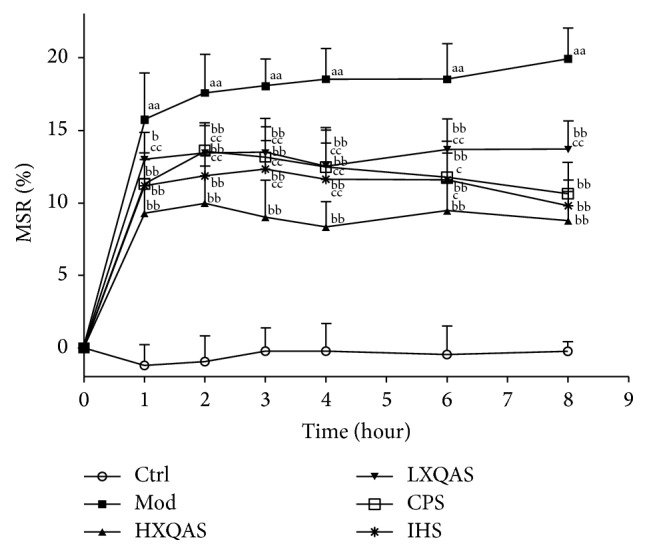
Effect of XQAS on muscle swelling rate (MSR) in the acute STI model induced by hitting hind leg muscle in rats. The rats were no modeling topically repeatedly treated with 150 *μ*L/time vehicles in the control (Ctrl), or modeling topically repeatedly treated with 150 *μ*L/time vehicles in the acute STI model (Mod), XQAS in the high-dose and low-dose XQAS-treated group (HXQAS and LXQAS, containing 0.50 or 0.25 g/mL both* CP* and* IH*, resp.), CPS (containing 1.0 g/mL* CP*) (CPS), or IHS (containing 1.0 g/mL* IH*) (IHS) for 8 h. The data were shown as mean ± SD (*n* = 8 per group). aa: *P* < 0.01 versus Ctrl; b: *P* < 0.05, or bb: *P* < 0.01 versus Mod. c: *P* < 0.05, or cc: *P* < 0.01 versus HXQAS.

The MSR in the hit hind leg of rats quickly elevated at 1 h after hitting and reached top extent at 2 h, which were in the top value always at 2 h to 8 h after hitting. XQAS treatment dose-dependently attenuated MSR with significant inhibition on MSR starting from 1 h after hitting in the HXQAS and LXQAS group, respectively. Either CPS or IHS treatment also attenuated MSR similarly to XQAS. However, the effect of HXQAS was obviously superior to both CPS effect at 2 h, 3 h, 4 h, and 6 h after hitting and IHS effect at 3 h, 4 h, and 6 h after hitting, exhibiting an effect with “one plus one more than two,” suggesting a synergistic effect between* CP* and* IH* on inhibition of hit-induced acute traumatic swelling (Figure 1).

### 3.2. Evaluation of XQAS on Anti-Inflammatory Reaction and Antioxidative Stress in the Acute STI Model Induced by Hitting Hind Leg Muscle in Rats

#### 3.2.1. Effects of XQAS on mRNA Expressions and Levels of TNF-*α* and IL-1*β* in the Injured Muscle Tissues

**Figure 2:**
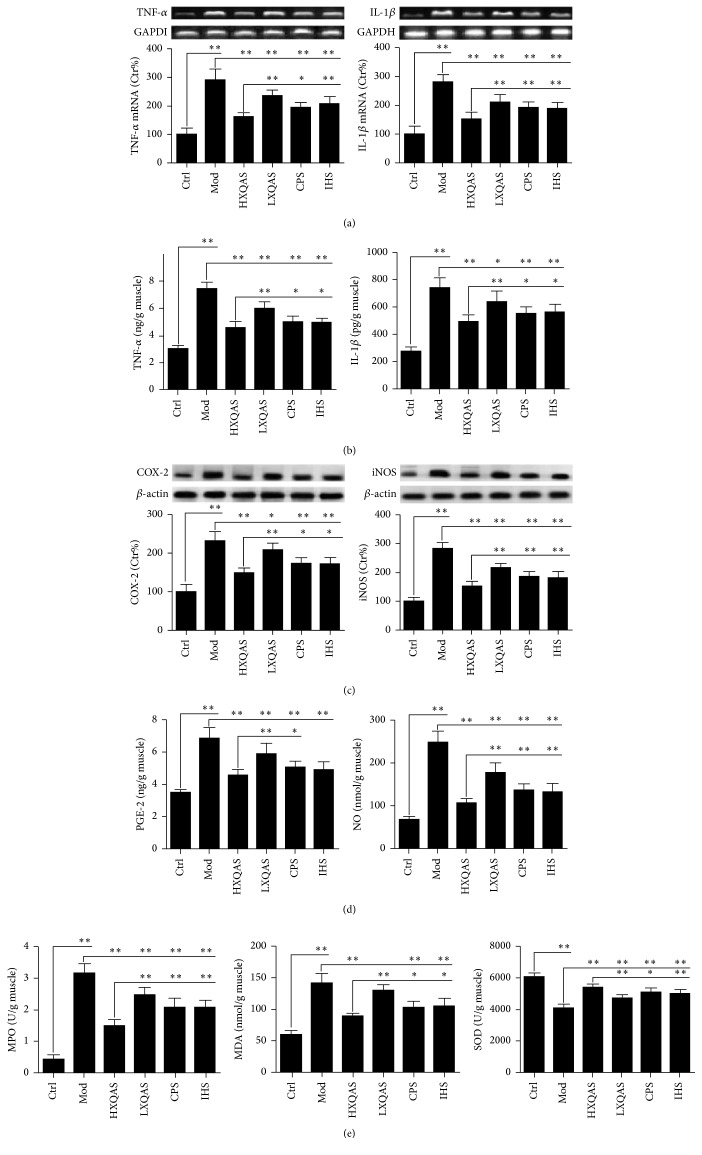
Effects of XQAS on inflammation-related agents with expressions of TNF-*α* ((a), left) and IL-1*β* ((a), right), mRNA and their levels (b), expressions of COX-2 ((c), left) and iNOS ((c), right), and levels of PGE-2 ((d), left) and NO ((d), right) and on oxidative stress-related agents with MPO activity ((e), left), MDA levels ((e), middle), and SOD activity ((e), right) in the control (Ctrl), acute STI model (Mod), high-dose XQAS-treated (HXQAS), low-dose XQAS-treated (LXQAS), CPS-treated (CPS), and IHS-treated (IHS) group after modeling with or without repeated drug treatment for 8 h. The data of mRNA or protein expression were shown as mean ± SD (*n* = 6 per group), while the data of biochemical indicators were shown as mean ± SD (*n* = 8 per group). ^*∗*^ *P* < 0.05; ^*∗∗*^ *P* < 0.01.

As inflammation is the most important reaction when acute STI occurs [19], we found that the mRNA expressions and levels of TNF-*α* and IL-1*β* in the damaged muscle tissue were markedly increased at 8 h after hitting hind leg muscle. XQAS treatment dose-dependently inhibited the overexpression of TNF-*α* and IL-1*β* mRNA and both levels increased in the acute injured muscle tissue. These effects were also observed in both CPS- and IHS-treated group but were significantly less than the HXQAS effects, indicating a synergistic effect between* CP* and* IH* (Figures 2(a) and 2(b)).

#### 3.2.2. Effects of XQAS on Expressions of COX-2 and iNOS and Levels of PGE-2 and NO in the Injured Muscle Tissue

The overexpressions of COX-2 and iNOS with increased PGE-2 and NO levels in the damaged muscle tissue were also exhibited. These abnormalities were suppressed markedly by XQAS treatment in a dose-dependent manner or by either CPS or IHS treatment, while the more significant suppression was found in the HXQAS-treated group compared to the CPS- or IHS-treated group, also suggesting a synergistic effect between both (Figures 2(c) and 2(d)).

#### 3.2.3. Effects of XQAS on Oxidative Stress in the Injured Muscle Tissue

Oxidative stress in the injured skeletal muscle is produced, which is known to accelerate progression of muscle pathologies [20] and is able to be marked by increased MPO activity and MDA level with decreased SOD activity [21–23], which were detected in the damaged muscle tissue in this study. XQAS, CPS, and HIS treatment obviously inhibited these changes and XQAS effects showed a dose-dependent manner. With an equal-dose treatment, the XQAS showed more effective effects than CPS or HIS on suppressing production of oxidative stress in the injured muscle, indicating a synergistic effect between both (Figure 2(e)).

### 3.3. Evaluation of XQAS on Inhibition of Inflammation Signaling Pathway Activated by Acute STI

#### 3.3.1. Effects of XQAS on Degradation of I*κ*B*α*, Expression of NF-*κ*B p65 mRNA, and Expressions of NF-*κ*B p65 in the Nucleus and Cytosol in the Injured Muscle

**Figure 3:**
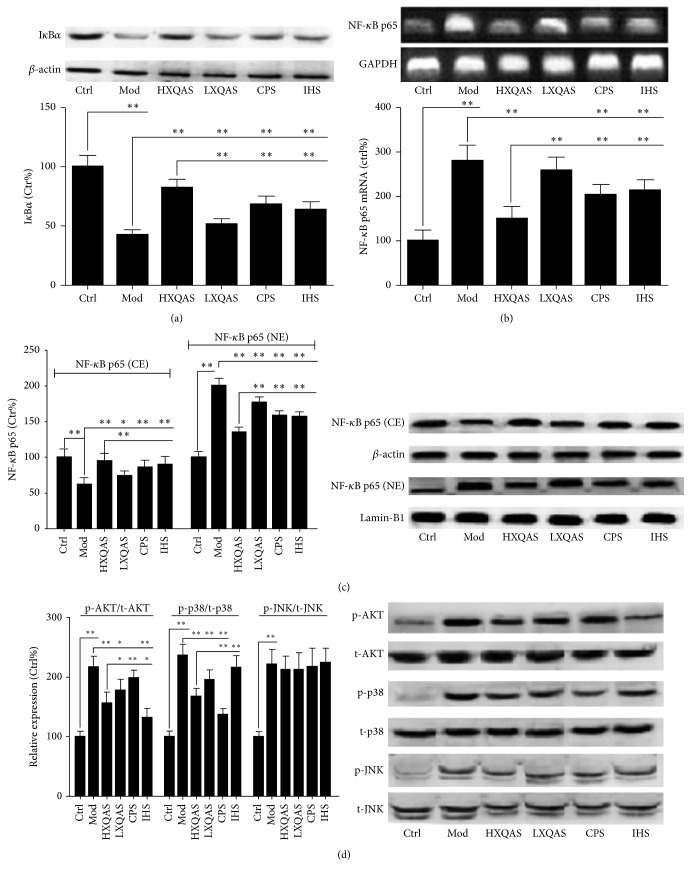
Effects of XQAS on inflammation-related signaling pathway with I*κ*B*α* expression in cytosol (a), NF-*κ*B p65 mRNA expression (b), NF-*κ*B p65 expression in cytosol extraction (CE) and nuclei extraction (NE) (c), and expressions of total AKT (t-AKT), Ser^473^ phosphorylation of AKT (p-AKT), total p38 (t-p38), Thr^180^/Tyr^182^ phosphorylation of p38 (p-p38), total JNK (t-JNK), and Thr^183^/Tyr^185^ phosphorylation of JNK (p-JNK) in the control (Ctrl), acute STI model (Mod), high-dose XQAS-treated (HXQAS), low-dose XQAS-treated (LXQAS), CPS-treated (CPS), and IHS-treated (IHS) group after modeling with or without repeated drug treatment for 8 h. The data were shown as mean ± SD (*n* = 6 per group). ^*∗*^ *P* < 0.05; ^*∗∗*^ *P* < 0.01.

NF-*κ*B is thought of as a genetic switch to control the inflammation-related target genes and proteins expression [14]. In this acute STI model, an activated inflammatory signaling pathway was exhibited with downregulated I*κ*B*α* expression in the cytosol, indicating that I*κ*B*α* was degraded in the cytosol, upregulated expression of NF-*κ*B p65 mRNA, and downregulated in the cytosol and upregulated in the nuclei NF-*κ*B p65 expression, indicating that NF-*κ*B p65 synthesis was enhanced and that more NF-*κ*B p65 was translocated to the nucleus. XQAS treatment dose-dependently inhibited hypoexpression of I*κ*B*α* in the cytosol, hyperexpression of NF-*κ*B p65 mRNA, and hypoexpression in the cytosol with hyperexpression in the nuclei of NF-*κ*B p65 in the injured muscle, suggesting that XQAS can inhibit acute STI-induced I*κ*B*α* degradation, overexpression of NF-*κ*B p65 gene, and NF-*κ*B p65 translocation into nuclei from cytosol, all of which results in an inhibition in activated inflammatory signaling pathway. Treatment with CPS or IHS alone, as an equal dose with XQAS, also showed similar effects with XQAS but inferior significantly to XQAS, indicating a synergistic effect between* CP* and* IH* on inhibiting acute STI-induced activation of inflammatory signaling pathway (Figures 3(a)–3(c)).

#### 3.3.2. Effects of XQAS on Activation of AKT, p38, and JNK in the Injured Muscle

Activation of MAPK (p-38, JNK, etc.) and AKT signaling cascadedly activate their downstream NF-*κ*B by promoting I*κ*B*α* degradation and NF-*κ*B gene expression [14]. In the present study, the levels of NF-*κ*B gene expression significantly increased, followed by NF-*κ*B expressions increased both in endochylema and in nucleus, while XQAS, CPS, and IHS treatment can inhibit NF-*κ*B expressions abnormal increase in different degrees. The levels of total AKT (t-AKT), total p38 (t-p38), and total JNK (t-JNK) expression were not changed markedly in the injured muscle, while the levels of phosphorylation of AKT (p-AKT), phosphorylation of p38 (p-p38), and phosphorylation of JNK (p-JNK) expression were significantly increased, suggesting AKT, p38, and JNK were activated. XQAS treatment dose-dependently inhibited the activations of both p-AKT and p-p38 in the acute STI model. Notably, CPS treatment masterly suppressed p38 activated but almost did not change AKT activated with only a decreased tendency in phosphorylated AKT expression. However, IHS treatment, showing a contrary action with CPS, masterly suppressed AKT activated but almost did not change p38 activated with very little decrease in phosphorylated p38 expression. In addition, comparing the effects of XQAS, CPS, and IHS with an equal-dose treatment on suppressing both AKT and p38 activated, the XQAS effect was less than the CPS on suppressing p38 activated and was also less than the IHS on suppressing AKT activated. Treatment with XQAS, CPS, or IHS did not improve p-JNK activated in the injured muscle (Figure 3(d)).

### 3.4. Histopathologic Evaluation of Acute Injured Muscle Tissue with or without XQAS, CPS, and IHS Treatment

**Figure 4:**
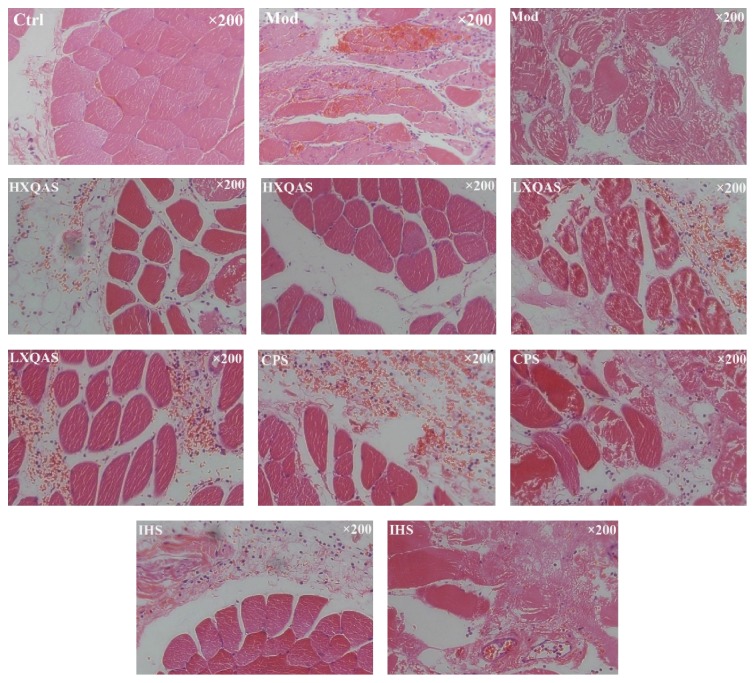
Representative histopathological micrographs (×200, HE staining) in the control (Ctrl), acute STI model (Mod), high-dose XQAS-treated (HXQAS), low-dose XQAS-treated (LXQAS), CPS-treated (CPS), and IHS-treated (IHS) group after modeling with or without repeated drug treatment for 8 h. Normal muscle tissue in Ctrl; severe muscle fibers disruption and necrosis, significant red blood cell accumulation in the interstitial space, and inflammatory cells infiltration in the Mod; very significant pathological improvement in injured muscle with markedly reduced area of the disruption and necrosis, decreased interstitial ecchymosed and inflammatory cells infiltration in the HXQAS group, and still marked muscle tissue injury but improved with marked muscle fibers disruption and necrosis, red blood cell accumulation in the interstitial space, and inflammatory cells infiltration in the LXQAS, CPS, and IHS group were shown.

In the Ctrl group, no marked injury was exhibited in the muscle tissue, while in the model group, severe muscle tissue injury was exhibited with large areas of muscle fibers disruption, muscle cells necrosis and significant vascular damage leading to red blood cell accumulation in the interstitial space, and considerable inflammatory cells infiltration in the interstitial space. In the HXQAS group, injured muscle was significantly improved with greater reduced muscle fibers disruption and necrosis and significantly decreased interstitial ecchymosed following a few inflammatory cells infiltration. In the LXQAS group, the muscle-injured improvement was less than that in the HXQAS group, with a marked interstitial ecchymosis, muscle cell necrosis, and inflammatory cells infiltration. In the CPS or IHS group, the injured muscle was also improved significantly but less than that in the HXQAS group. Representative histopathological findings in each group are shown in Figure 4.

## 4. Discussion

The early recovery phase of STI is characterized by the overlapping processes of inflammation and following occurrence of secondary damage [4]. Within the injured muscle tissue there is leukocyte infiltration and local production of various pro- and anti-inflammatory cytokines, which are crucial for initiating the breakdown and the subsequent removal of damaged muscle fragments, but overexpression of proinflammatory cytokines can be infaust to subsequent healing [24]. In our present study, it was found that the proinflammatory cytokines and their gene overexpressions in the acute STI muscle tissue were notably suppressed by XQAS treatment, which demonstrates that XQAS has an efficacy to inhibiting inflammatory injuries in the acute closed STI. XQAS had quick treatment effects on acute STI, and the active components contained in the* CP* and* IH*, which compose XQAS formulation, have a synergistic effect on treating acute STI.

Free radical generation is known to accelerate progression of muscle pathologies [20]. Oxidative stress in skeletal muscle is increased during acute STI and is ascribed to increases in enzyme-initiated oxidant production and neutrophil-derived myeloperoxidase (MPO) activity [21]. In the present study, XQAS displayed a strong effect on reduction of MPO activity as well as attenuation of inflammatory cell infiltration in the interstitial space of injured muscle. Meanwhile, increased MDA level and decreased SOD activity, both of which are markers of oxidative stress [22], were significantly reversed by XQAS in a dose-dependent manner. Cyclooxygenase (COX), an enzyme that converts arachidonic acid to PGs, has been found to have two isoforms, namely, COX-1 and COX-2. COX-2 is responsible for production of large amounts of proinflammatory PGs, especially PGE-2, at the inflammatory site [25]. PGE-2 has an effect on dilating blood vessels, resulting in fever and pain. It can also work synergistically with other inflammatory mediators to exacerbate inflammatory response [26]. NO, which is produced by iNOS activation, can regulate contractile function of skeletal muscle [27] and also lead to vasodilatation to increase perfusion, which is essential for remodeling the damaged muscle but results in tissue edema [28]. XQAS can also suppress STI-caused upregulations of COX-2 and iNOS expression with overproductions of PGE-2 and NO in a dose-dependent manner. In addition, the effects of treatment with CPS and IHS alone on improving aforementioned abnormality in the injured muscle tissue were less than the XQAS effects and there is a synergistic effect between* CP* and* IH* components in the XQAS.

NF-*κ*B is a pivotal factor that transfers inflammatory signals from cytoplasm into the nucleus and induces a series of inflammatory responses in the cell [29]. During inactive state, NF-*κ*B subunit p65/p50 is combined with its multiple inhibitors, I*κ*Bs such as I*κ*B*α*, in the cytoplasm. I*κ*Bs can be phosphorylated by I*κ*B kinase (IKK*α* and IKK*β*) resulting in separation between I*κ*B and NF-*κ*B and I*κ*B degradation via ubiquitination; the nuclear localization signal of NF-*κ*B located in the p65 is then unmasked. Next, the NF-*κ*B complex would translocate into the nucleus after separation with I*κ*Bs [14]. NF-*κ*B activity can be regulated by MAPK and AKT pathway activation through promoting degradation of I*κ*B*α*. The MAPK family consists of extracellular signal-regulated kinases 1/2 (ERK1/2), c-Jun N-terminal kinase (JNK), and p38 [30]. p38 not only leads to I*κ*B*α* phosphorylation but also leads to nuclear p65 phosphorylation at Ser^276^ via activating mitogen- and stress-activated protein kinase-1 (MSK-1) [31]. However, JNK mainly mediates NF-*κ*B signaling via effects on c-Jun and c-Fos [32]. AKT mediates NF-*κ*B activity mainly through inducing IKK*β* phosphorylation and then leads to I*κ*Bs ubiquitinated degradation [33]. An important aspect of the significance of NF-kB p65/p50 in inflammatory reaction stems from it both being activated by and inducing the expression of inflammatory cytokines [14].

A recent study has indicated that blockade of MAPK-NF-*κ*B pathway activation protects mice from tissue injury by reducing the production of proinflammatory cytokines [34]. Suppression of AKT activation has also been shown to reduce LPS-induced inflammatory responses in the human endothelial cells [35]. From our results, we found that XQAS by suppressing activations of both AKT and p38 pathway quickly abolished activation of NF-*κ*B p65. NF-*κ*B gene expression and its translocation from cytosol to nucleus were enhanced owing to I*κ*B*α* degraded in the acute injured muscle, while XQAS treatment led to a strong suppression on inflammatory cascade to acute STI. XQAS combining characteristics of CPS and IHS, where CPS merely suppressed p38 activation while IHS suppressed AKT activation majorly, achieved a super effectiveness on preventing I*κ*B degraded and NF-*κ*B translocation into nucleus activated by STI. There is a synergistic effect between CPS and IHS active components in the XQAS. The critical super effectiveness of XQAS on injured muscle may be effective suppression on I*κ*B*α* degradation to lead to abolishment of NF-*κ*B translocation from cytosol to nucleus, resulting in a strong suppression on transcriptional activation of NF-*κ*B itself and target with inflammation-related genes and on subsequent muscle necrosis, blood stasis in the interstitial space, inflammatory cells infiltration, oxidative stress, and acute traumatic swelling. In addition, though JNK was also activated in the injured muscle tissue, XQAS, CPS, and IHS all did not alter the state of JNK activation.

Although inflammation is generally considered as a normal and necessary prerequisite to injury recovery [36, 37], it is indeed detrimental when inflammation cascade gets out of control, such as restricting the blood flow due to increased local hydrostatic pressure in the oedema site, pain, and reducing local oxygen levels, during acute STI [38]. Both our present study and previous study [16] indicated that instant suppression to acute STI-induced excess inflammation in the initiation of acute STI is very beneficial for prevention from secondary damage to normal muscle cells and muscle necrosis with subsequent degenerative processes to lead to muscle tissue replacement by excess fibrin deposits. The impressive effects of XQAS on treating STI probably owe to its very effective suppression to inflammatory cascade in the initiation of STI.

## 5. Conclusion

**Figure 5:**
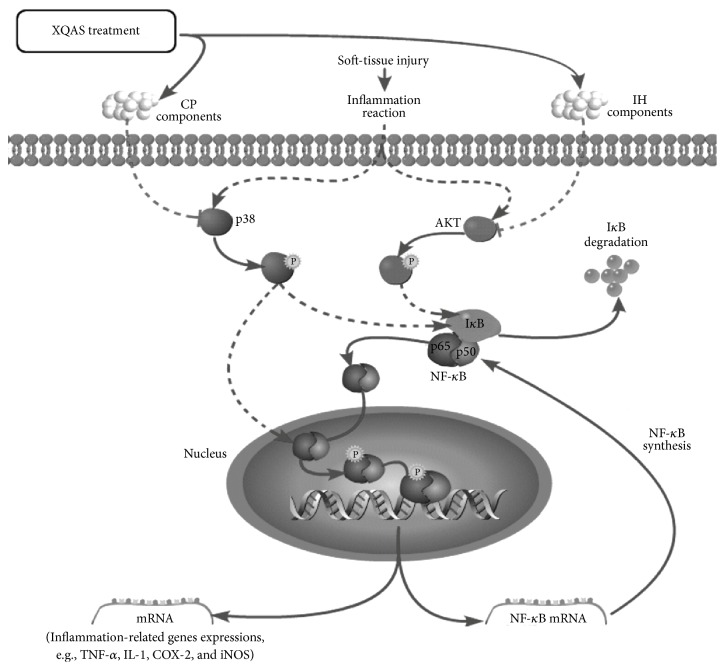
Mechanism of XQAS effects on blocking inflammation-signal pathway in the injured muscle tissue.

XQAS is a quite reasonable formulation with a combination of active components of* Cynanchum paniculatum* (*CP*) and* Illicium henryi* (*IH*) in treating acute STI. The mechanism of impressive effects of XQAS, at least majorly, is due to its anti-inflammatory and antioxidative activities with a synergistic effect between active components of* CP* and* IH*, in which the* CP* components suppress STI-induced p38 phosphorylated activation with intranuclear NF-*κ*B p65 phosphorylation, while the* IH* components suppress STI-induced AKT phosphorylated activation; subsequently, I*κ*B degradation in the cytoplasm, which is promoted by both p38 and AKT pathways activation, is blocked to lead to decrease in NF-*κ*B p65/p50 translocated into the nucleus after separation with I*κ*B; ultimately, NF-*κ*B p65/p50 actions on transcriptional activation of NF-*κ*B itself and target with inflammation-related genes, all of which are enhanced by both p38 and AKT pathways activation, are abolished. The mechanism process was shown in Figure 5.
